# An Accuracy Assessment: Responses to *Mycoplasma Pneumoniae* Pneumonia‐Related Questions by Different Artificial Intelligence Tools

**DOI:** 10.1111/crj.70005

**Published:** 2024-08-26

**Authors:** Shuang Li

**Affiliations:** ^1^ Department of General Practice of the Second Affiliated Hospital of Fujian Medical University Quanzhou China

**Keywords:** artificial intelligence, ChatGPT, ERNIE Bot, Kimi, Mycoplasma Pneumoniae Pneumonia

With the rapid development of socio‐economic technology, artificial intelligence (AI) is increasingly applied in daily life. When discussing AI, it is inevitable to mention ChatGPT, a language model based on AI technology. Pretrained on extensive language data, ChatGPT can perform various natural language processing tasks, including dialog generation. In addition, similar large language models include the intelligent assistant Kimi launched by Beijing Lunar Tech and the chatbot ERNIE developed by Baidu, among others.

*Mycoplasma pneumoniae* pneumonia, caused by infection with *Mycoplasma pneumoniae* , refers to inflammation of the lungs that can affect the bronchi, bronchioles, alveoli, and interstitial tissue. It can occur at any age but is more common in children aged 5 and above, as well as in immunocompromised individuals (such as the elderly, immunodeficient individuals, or patients undergoing immunosuppressive therapy). The course of *Mycoplasma pneumoniae* pneumonia is generally 1–2 weeks, with a favorable prognosis and no sequelae in most cases. However, a small number of cases can develop into severe conditions, primarily presenting with symptoms of respiratory distress and respiratory failure. These severe cases are often associated with acute respiratory distress syndrome, plastic bronchitis affecting the large airways, diffuse bronchiolitis, and severe pulmonary embolism. In rare instances, severe extrapulmonary complications may be the main manifestations. Given the prevalence of *Mycoplasma pneumoniae* infection in China, we sought to understand whether ChatGPT could contribute to a better understanding of *Mycoplasma pneumoniae* pneumonia. We selected 13 questions that are most commonly asked by patients in clinical practice and posed them to ChatGPT‐3.5, ChatGPT‐4.0, Kimi, and ERNIE. Each question was run five times. Then we invited nine experts with extensive clinical experience and knowledge of Mycoplasma pneumonia to rate the accuracy of the answers. Among them, there were four respiratory specialists and five pediatric specialists, with five from our hospital and four from other hospitals. The scoring criteria were as follows: score = 0: completely incorrect; score < 6: inaccurate; 6 ≤ score < 8: mostly accurate; 8 ≤ score < 10: very accurate; score = 10: completely accurate. The final results were ChatGPT 3.5 8.46 ± 0.80, ChatGPT 4.0 7.05 ± 1.16, Kimi 8.62 ± 0.68, and ERNIE 9.33 ± 0.30. In descending order of accuracy, they were ERNIE, Kimi, ChatGPT 3.5, and ChatGPT 4.0.

We compared the answers provided by ChatGPT versions 3.5 and 4.0, ERNIE, and Kimi regarding Mycoplasma pneumonia and found that their accuracy ranked from the highest to the lowest as ERNIE, Kimi, ChatGPT 3.5, and ChatGPT 4.0, with ERNIE achieving the highest accuracy. Although ERNIE performed the best among the four AI models with more comprehensive answers, it still exhibited some answers with noticeable errors. For instance, in question 11 regarding treatment options for Mycoplasma pneumonia, ERNIE suggested that penicillin antibiotics have some effectiveness against Mycoplasma pneumonia. It is widely known that *Mycoplasma pneumoniae* lacks a cell wall, rendering antibiotics targeting cell walls ineffective unless bacterial co‐infection is present. Another example is in question 2 on how *Mycoplasma pneumoniae* spreads, where ERNIE's answer suggested sexual transmission, which is not supported by evidence found in relevant PubMed literature.

Surprisingly, when we compared ChatGPT versions 3.5 and 4.0, we found that despite OpenAI's claims that ChatGPT 4.0 surpasses ChatGPT 3.5 in language understanding, generation capabilities, and performance, ChatGPT 3.5 consistently received higher scores for the questions we posed about Mycoplasma pneumonia. Clearly, in terms of accuracy, ChatGPT 3.5 outperformed ChatGPT 4.0. Furthermore, we observed that compared to ChatGPT 3.5, ChatGPT 4.0, while more vivid and engaging with fewer technical terms, often provided less comprehensive and specific answers, occasionally leading to misunderstandings. For example, in question 1 about what Mycoplasma pneumonia is, ChatGPT 4.0's answer contained factual errors regarding chest radiographs.

Furthermore, upon comparing the answers from the four AI tools with the latest guidelines, we found instances where the AI answers were inaccurate or incomplete, in addition to the issues mentioned above. For example, regarding question 8, the answers from the four AI tools regarding complications of *Mycoplasma pneumoniae* pneumonia are not comprehensive. The course of *Mycoplasma pneumoniae* pneumonia is generally 1–2 weeks, with a favorable prognosis and no sequelae in most cases. However, a small number of cases can develop into severe conditions, primarily presenting with symptoms of respiratory distress and respiratory failure. These severe cases are often associated with acute respiratory distress syndrome, plastic bronchitis affecting the large airways, diffuse bronchiolitis, and severe pulmonary embolism. In rare instances, severe extrapulmonary complications may be the main manifestations. However, the answers from all four AI tools overlooked plastic bronchitis and pulmonary embolism [1]. Plastic bronchitis is a significant contributor to severe and fulminant *Mycoplasma pneumoniae* pneumonia, presenting with persistent high fever, respiratory distress, and physical examination findings such as tracheal casts, subcutaneous emphysema, and decreased or absent breath sounds in the lungs [1]. Pulmonary embolism may occur independently or concurrently with emboli in other locations, serving as a cause of necrotizing pneumonia and a significant factor in residual lung atelectasis and organizing pneumonia [1].

For question 11 concerning the drug treatment of *Mycoplasma pneumoniae* pneumonia, the answers from the four AI tools are not sufficiently accurate. A systematic review and meta‐analysis have shown a global trend of increasing macrolide‐resistant *Mycoplasma pneumoniae* infections, rising from 18.2% in 2000 to 76.5% in 2019 [2]. The highest infection rates are observed in the Western Pacific region, with mainland China accounting for 79.5% [2]. Additionally, a multicenter study in China from 2013 to 2019 indicated that macrolide resistance in *Mycoplasma pneumoniae* increased from 75.8% to 97.4% [3]. Therefore, Chinese guidelines recommend macrolide antibiotics as the first‐line treatment for *Mycoplasma pneumoniae* pneumonia in children [1]. For cases resistant to macrolides, children over 8 years old may be treated with newer tetracyclines, which can potentially cause tooth discoloration and enamel hypoplasia, necessitating careful risk–benefit assessment for children under 8 years old. Fluoroquinolone antibiotics are used as alternative therapies for suspected or confirmed cases of MUMPP (macrolide‐unresponsive *Mycoplasma pneumoniae* pneumonia), RMPP (refractory *Mycoplasma pneumoniae* pneumonia), or SMPP (severe *Mycoplasma pneumoniae* pneumonia), despite the risk of cartilage injury in animals and tendon rupture in humans, requiring thorough risk assessment for individuals under 18 years old. In adults [4], due to high macrolide resistance rates, oral doxycycline or minocycline are preferred for Mycoplasma infections. Macrolides may be empirically used in regions with low resistance rates. Respiratory fluoroquinolones are used as alternative treatments in areas with high drug resistance rates or for patients allergic to or intolerant of other medications. Besides antimicrobial therapy, symptomatic treatments such as antipyretics, cough suppressants, and expectorants are also employed. Severe cases may require glucocorticoids or bronchoalveolar lavage. Clearly, the four AI tools did not specify the differences in antimicrobial treatment between children and adults, which may lead to incorrect use, ineffective treatment, or exacerbation of conditions.

In summary, although AI technology is advancing rapidly with the emergence of various large‐scale language models such as ChatGPT, Kimi, and ERNIE, answers to questions related to *Mycoplasma pneumoniae* pneumonia are still often incomplete, inaccurate, or even clearly erroneous. This may be due to training data not being updated in accordance with the latest guidelines. Furthermore, the application of medications must consider various factors such as patient age, past medication history, and drug side effects. AI tools typically provide lists of treatment medications and may not offer personalized treatment plans based on individual patient conditions. Therefore, while AI tools can be helpful for addressing questions related to *Mycoplasma pneumoniae* pneumonia, final decisions should still rely on healthcare professionals, as every answer ultimately concludes with “Consult a healthcare professional if you have related concerns.”

Although AI tools have limitations, overall, their accuracy in answers is relatively high. Such tools can explain common health issues to patients, to some extent saving healthcare resources, while also promoting medical knowledge and aiding in doctor–patient communication and shared decision‐making. We believe that with ongoing efforts from researchers, AI may become more specialized and humane in its applications in the medical field in the future.

## Author Contributions

Shuang Li designed the study and completed the data collection and manuscript writing.

## Disclosure

Permission to reproduce material from other sources has been obtained and is acknowledged accordingly.

## Ethics Statement

The author has nothing to report.

## Consent

The author has nothing to report.

## Conflicts of Interest

The author declares no conflicts of interest.

## Supporting information


**Appendix S1** Supplementary Information

## Data Availability

The data supporting the findings of this study are available upon reasonable request.
